# A structural study of 2,4-di­methyl­aniline derivatives

**DOI:** 10.1107/S2056989018011404

**Published:** 2018-08-21

**Authors:** Samuel C. Penner, Maryam Kalvandi, Jamie S. Ritch

**Affiliations:** a Department of Chemistry, The University of Winnipeg, Winnipeg MB R3B 2E9, Canada

**Keywords:** crystal structure, reactivity studies, hydrogen bonding, anilines, piperazines

## Abstract

Crystallographic studies of nitro­gen-containing small mol­ecules aid in the elucidation of their structure–activity relationships and modes of aggregation. In this study, two previously synthesized mol­ecules are crystallographically characterized for the first time. Reaction of 2,4-di­methyl­aniline with *N*-bromo­succinimide affords the *ortho*-brominated derivative 2-bromo-4,6-di­methyl­aniline (**1**; C_8_H_10_BrN), which sublimates *in vacuo* to afford crystals featuring hydrogen-bonded chains as well as Type I halogen–halogen inter­actions. Conversely, alkyl­ation of two equivalents of 2,4-di­methyl­aniline with 1,2-di­bromo­ethane affords a separable mixture of *N*,*N*′-bis­(2,4-di­methyl­phen­yl)piperazine (**2**; C_20_H_26_N_2_), which was crystallographically characterized, as well as *N*,*N*′-bis­(2,4-di­methyl­phen­yl)ethyl­enedi­amine (**3**).

## Abstract 2

The crystal structures of two aromatic amines are presented: a hydrogen-bonded brominated aniline, and a piperizine derivative.

## Chemical context   

Anilines are important building blocks for value-added chemicals such as indoles, which feature prominently in therapeutic agents (Humphrey & Kuethe, 2006 ▸). Polyaniline, formed by oxidative coupling of aniline, is a valuable conductive polymer used in advanced materials research (Kang *et al.*, 1998 ▸). As they are prone to engage in hydrogen bonding, anilines have also been utilized in crystal engineering studies (Mukherjee *et al.*, 2014 ▸). The piperazine functional group is present in a number of active pharmaceutical ingredients. In particular, the widely used anti­fungal agent itracona­zole (Grant & Clissold, 1989 ▸), and anti­bacterial ciprofloxacin (Hooper & Wolfson, 1991 ▸) feature piperazine structural units with aryl-group substitution. We have an inter­est in constructing *N*-heterocyclic carbenes (NHCs) and NHC-derived ligands, which often feature *N*-aryl groups derived from substituted anilines. Halogenated NHCs can be utilized to fine-tune the steric and electronic properties of transition metal catalysts. It has been demonstated that the presence of fluorine on an aryl group of an NHC ligand influences the *E*/*Z* selectivity of a ruthenium cross-metathesis catalyst (Xu *et al.*, 2017 ▸). In our efforts to prepare NHC ligands, anilines and *N*,*N′*-di­aryldi­amines are commonly used starting materials or synthetic inter­mediates.

In this study, we report the crystallographic characterization of two compounds derived from 2,4-di­methyl­aniline: 2-bromo-4,6-di­methyl­aniline (**1**) and *N*,*N′*-bis­(2,4-di­methyl­phen­yl)piperazine (**2**). Though available from many commercial suppliers, the crystal structure of 2-bromo-4,6-di­methyl­aniline (**1**) has not been previously disclosed. Only a few reports of compound **2** can be found in the literature. An early publication (Tikhomirova, 1971 ▸) describes the reaction of 2-(2,4-di­methyl­anilino)ethanol with pyridinium chloride, which generates a mixture of 2,4-di­methyl­aniline and the piperazine **2**, which was characterized only by elemental analysis, melting point, and boiling point. More recently, piperazine **2** was obtained as a trace by-product in the production of 2-(2,4-di­methyl­anilino)ethanol *via* palladium-mediated hydrogen autotransfer between 2,4-di­methyl­aniline and ethyl­ene glycol (Llabres-Campaner *et al.*, 2017 ▸), and characterized by NMR and IR spectroscopy in addition to high resolution mass spectrometry. No X-ray structural data for compound **2** have been previously disclosed.
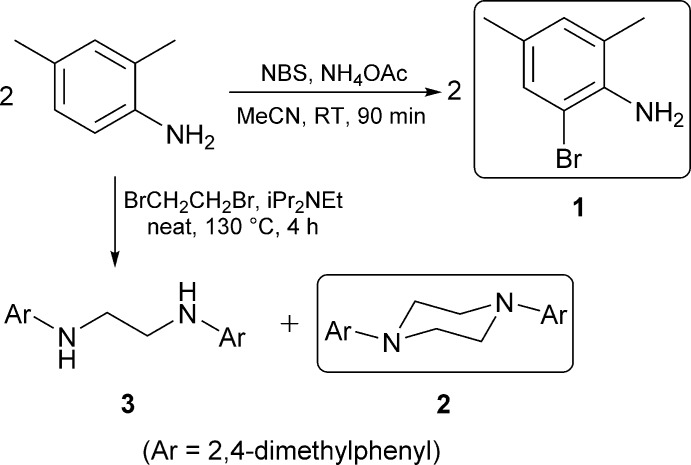


## Structural commentary   

**Figure 1:**
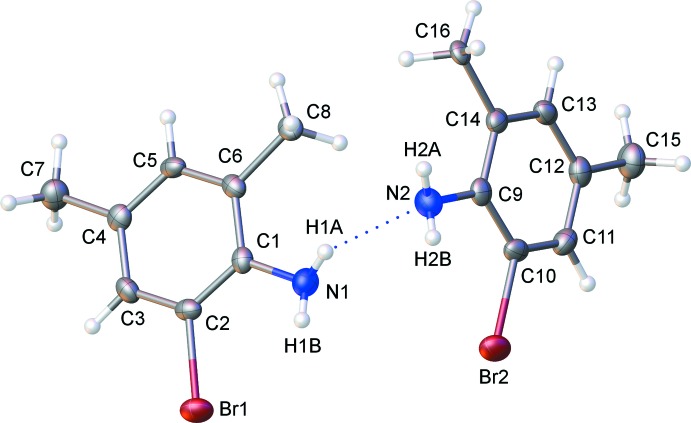
Displacement ellipsoid plot (50% probability) of the asymmetric unit of compound **1**.

The solid-state structure of **1** obtained by slow sublimation is depicted in Fig. 1 ▸. Two independent mol­ecules are present in the asymmetric unit, which are hydrogen bonded (Table 1 ▸) to each other [*d*(N⋯N) = 3.172 (5) Å] within the *P*2_1_/c space group. The two independent mol­ecules exhibit C—Br and C—N bond lengths that are equal within experimental error [1.910 (3)–1.912 (3) Å and 1.394 (4)–1.394 (5) Å, respectively]. The angle between the two mean planes passing through the aromatic rings of the two independent molecules is 80.6 (2)°. The hydrogen atoms on each nitro­gen centre that are not involved in the chains formed by the N—H⋯N interactions are oriented towards the *ortho* bromine atoms on the aromatic rings. These intra­molecular hydrogen bonds feature donor-acceptor distances of 3.082 (4) and 3.094 (4) Å.

**Figure 2:**
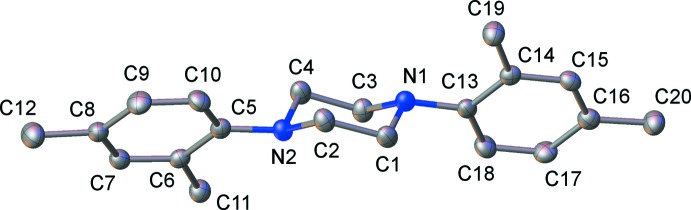
Displacement ellipsoid plot (50% probability) of the asymmetric unit of compound **2**. Hydrogen atoms are omitted for clarity.

X-ray diffraction analysis of **2** revealed a solvent-free structure in the *P*2_1_ space group (Fig. 2 ▸). The asymmetric unit contains one pseudo-*C*_i_ symmetric mol­ecule. The central N_2_C_4_ ring exhibits a chair conformation. Compound **2** represents the first crystallographically characterized di­aryl­piperazine with methyl groups on the aromatic substituents. The aromatic rings are twisted relative to the N_2_C_4_ mean plane, forming angles of 46.8 (1) and 56.9 (1)° for C5–C10 and C13–C18, respectively.

## Supra­molecular features   

**Figure 3:**
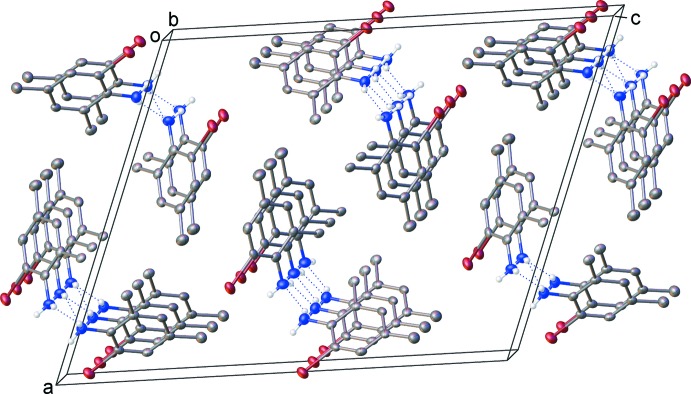
Packing diagram for compound **1**. Carbon-bound hydrogen atoms are omitted for clarity.

**Figure 4:**
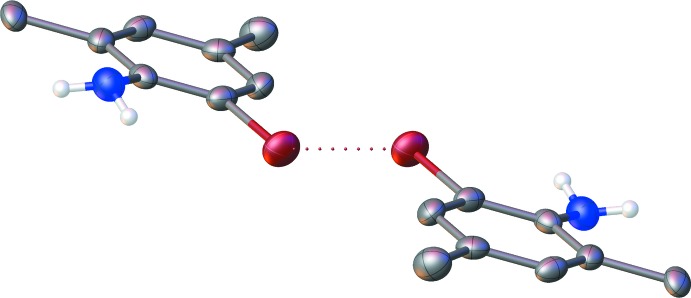
Type I bromine-bromine inter­action in the packing of compound **1**. Carbon-bound hydrogen atoms are omitted for clarity.

**Figure 5:**
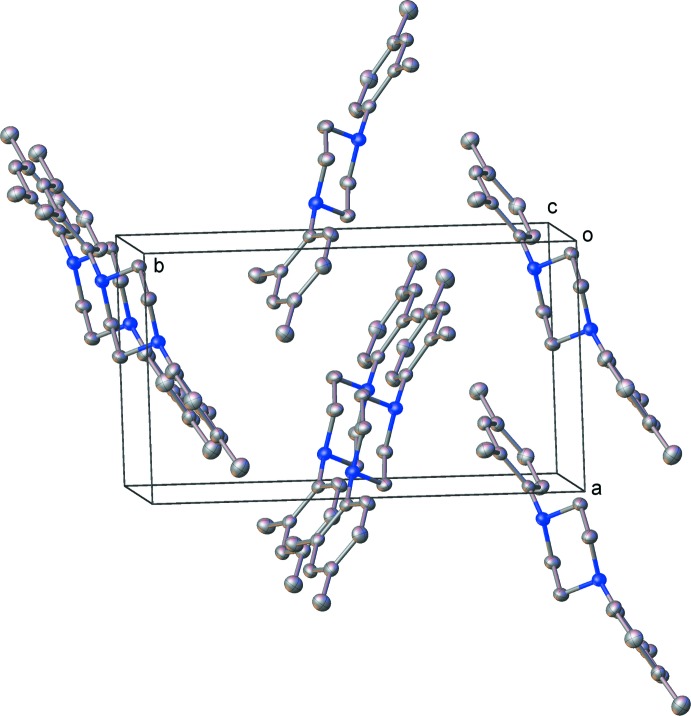
Packing diagram for compound **2**. Hydrogen atoms are omitted for clarity.

Each amino group in compound **1** provides one donor and one acceptor site for the hydrogen-bond inter­actions (Table 1 ▸), and chains are observed to form by translation along the crystallographic *b* axis (Fig. 3 ▸). Additionally, the bromine atoms from one of the two independent mol­ecules exhibit weak van der Waals inter­actions to the equivalent sites on adjacent chains, related by an inversion centre (Fig. 4 ▸). The distance for this inter­action is 3.537 (1) Å (sum of van der Waals radii for bromine: 3.70 Å; Bondi, 1964 ▸). As the two C—Br⋯Br bond angles are equal (*ca* 153 °), this classifies as a Type I halogen–halogen inter­action (Cavallo *et al.*, 2016 ▸). This type is generally accepted as a dispersion inter­action, as opposed to Type II inter­actions which are weakly electrostatic in nature and require *R*—*X*⋯*X* angles of 90 and 180°. No π–π inter­actions are present in the structure. No significant inter­molecular inter­actions are observed in the crystal packing motif of **2** (Fig. 5 ▸).

## Database survey   

The packing motif of compound **1** makes an inter­esting contrast to the structure of the less substituted analogue 2-bromo­aniline, which crystallizes from the melt in the trigonal *P*3_1_ space group (Nayak *et al.*, 2009 ▸). Helical arrangements are formed with each mol­ecule involved in inter­molecular N—H⋯N hydrogen bonds [*D*⋯*A* distance of 3.162 (6) Å] and a weaker bromine⋯bromine inter­action (Br⋯Br distance of 3.637 (1) Å), both observed along the 3_1_ screw axes, and with additional intra­molecular N–H⋯Br inter­actions. In the case of the more sterically hindered deriv­ative **1**, this arrangement is not feasible and chains are instead adopted.

**Figure 6:**
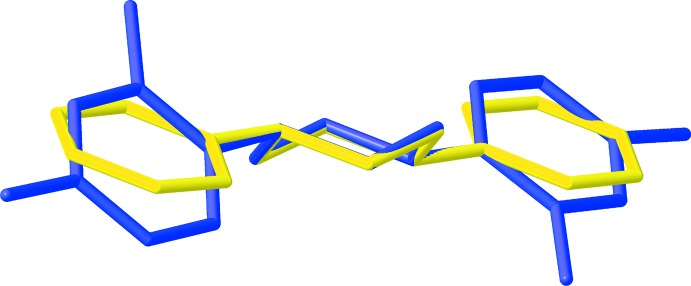
Structure of compound **2** overlaid with *N*,*N*′-di­phenyl­piperazine (CCDC refcode WAQNUZ01).

Most of the crystallographically characterized di­aryl­piperazines feature the chair conformation; a few have been determined in the twist-boat form (Wirth *et al.*, 2012 ▸). Whereas the phenyl groups of piperazine **2** are twisted relative to the N_2_C_4_ mean plane, the structure of the less substituted *N*,*N*′-di­phenyl­piperazine, which crystallizes in the *Pbca* space group, exhibits phenyl groups closer to being in conjugation with the nitro­gen lone pairs (Wirth *et al.*, 2012 ▸; Safko & Pike, 2012 ▸). The sum of the bond angles around nitro­gen is quite similar between the two structures (**2**: 338–341°; *N*,*N*′-di­phenyl­piperazine: 343°), though the N–C_ar­yl_ bond lengths are slightly shortened in the phenyl-substituted analogue [**2**: 1.426 (3)–1.431 (3) Å; *N*,*N*′-di­phenyl­piperazine: 1.4157 (15) Å], indicating that resonance delocalization is a perhaps a minor effect, if present, while packing effects likely dominate. The structures of **2** and the phenyl analogue are overlaid in Fig. 6 ▸ for visual comparison.

## Synthesis and crystallization   

We prepared compound **1** by electrophilic aromatic bromination of the parent aniline, as reported previously for related compounds (Das *et al.*, 2007 ▸). The resultant red-brown solid was reasonably pure by ^1^H NMR, however it was easily sublimated to afford very pure colourless material, leaving behind oily reddish-brown impurities.

The piperazine compound **2** was unexpectedly obtained as a by-product during the synthesis of *N*,*N*′-bis­(2,4-di­methyl­phen­yl)ethyl­enedi­amine (**3**) via a condensation reaction. Compound **3** is evidently able to compete with 2,4-di­methyl­aniline as a nucleophile towards 1,2-di­bromo­ethane, once formed. Both desired main product **3** and by-product **2** were isolated after separation by column chromatography.

Synthetic protocols were conducted under ambient conditions using ACS-grade solvents. All chemicals were obtained from commercial sources and used as received. NMR spectra were collected using a Bruker 400 MHz Avance III spectrometer. ^1^H and ^13^C resonances are referenced to residual CHCl_3_ or CDCl_3_, respectively, using the reported values relative to SiMe_4_ (Fulmer *et al.*, 2010 ▸).

### Preparation of 2-bromo-4,6-di­methyl­aniline (1)   

A 100 mL round-bottom flask equipped with a magnetic stir bar was charged with *N*-bromo­succinimide (3.4896 g, 19.607 mmol), ammonium acetate (0.1583 g, 2.054 mmol), and aceto­nitrile (60 mL). The reagent 2,4-di­methyl­aniline (2.4297 g, 20.050 mmol) was added slowly, by pipette. The resulting mixture was left to stir at room temperature for 90 min. The solvent was removed under vacuum to produce a reddish-brown solid. Water (45 mL) and di­chloro­methane (45 mL) were added, and the mixture was transferred to a separatory funnel. The organic layer was separated and washed with water (3 × 30 mL), saturated sodium thio­sulfate (30 mL), and brine (30 mL). After drying the organic layer with magnesium sulfate, the mixture was filtered and the volatiles removed under vacuum to afford a brown crystalline solid (3.2723 g, 83.42%). ^1^H NMR (CDCl_3_, 400 MHz): δ 7.15 (*s*, 1H), 7.14 (*s*, 1H), 3.93 (*s*, 2H), 2.22 (*s*, 3H), 2.21 (*s*, 3H). The procedure was based on one reported for similar aniline derivatives (Das *et al.*, 2007). The product can be purified by sublimation under static vacuum with heating to 308 K for 3 d. Large X-ray quality crystals of the product were obtained by slow sublimation under ambient conditions in a capped glass vial containing the crude product, over a period of months.

### Preparation of *N*,*N*′-bis­(2,4-di­methyl­phen­yl)piperazine (2) and *N*,*N*′-bis­(2,4-di­methyl­phen­yl)ethyl­enedi­amine (3)   

A 100 mL round-bottom flask equipped with a magnetic stir bar was charged with 2,4-di­methyl­aniline (9.21 mL, 74.5 mmol), 1,2-di­bromo­ethane (3.21 mL, 37.3 mmol), and *N*,*N′*-diiso­propyl­ethyl­amine (12.98 mL, 74.5 mmol), and fitted with a reflux condenser and drying tube. The mixture was heated to 403 K for 4 h, then cooled to room temperature affording a red solid mass. To this was added H_2_O (50 mL) before extraction with CH_2_Cl_2_ (30 mL). The organic phase was washed with H_2_O (50 mL), and to the combined aqueous extracts was added 1 *M* NaOH(aq) (40 mL), and this mixture was extracted with CH_2_Cl_2_ (50 mL). The combined organic extracts were washed with H_2_O (30 mL) and brine (30 mL), dried over MgSO_4_, deca­nted into a round-bottom flask, and dried under vacuum to afford a dark orange–red liquid. Addition of hexa­nes (40 mL) resulted in the precipitation of crystalline material. The solid material was redissolved by warming the hexa­nes, and the resultant clear red solution was stored overnight at 238 K. The mother liquor was deca­nted and the remaining solid material was washed with cold hexa­nes (3 × 3 mL) and dried under vacuum to afford a beige solid (5.4730 g). NMR data indicated that the product was a 90:10 mol% mixture of 1,2-di­amine **3** and piperazine **2**, obtained with a 69% yield of products based on 1,2-di­bromo­ethane. Separation of the compounds was achieved by silica gel flash chromatography. Elution of 1.3223 g of a mixture with CH_2_Cl_2_ afforded piperazine **2** as a pale-tan crystalline solid (*R*_f_ = 0.75, 124.0 mg, 60% recovery) and di­amine **3** as a pale-yellow solid (*R*_f_ = 0.21, 851.4 mg, 84% recovery). The ^1^H and ^13^C chemical shifts and assignments for di­amine in CDCl_3_ differed from the reported values (Türkmen & Çetinkaya, 2006 ▸). Di­amine **3**: ^1^H NMR (CDCl_3_): δ 6.96 (*d*, ^3^ *J*_HH_ = 8.1 Hz, 2H, aromatic 5-H), 6.91 (*s*, 2H, aromatic 3-H), 6.61 (*d*, ^3^ *J*_HH_ = 8.1 Hz, 2H, aromatic 6-H), 3.61 (*br s*, 2H, NH), 3.47 (*s*, 4H, CH_2_), 2.25 (*s*, 6H, aromatic 4-CH_3_), 2.11 (*s*, 6H, aromatic 2-CH_3_). ^13^C{^1^H} NMR (CDCl_3_): δ 144.0 (*s*), 131.3 (*s*), 127.6 (*s*), 126.8 (*s*), 122.8 (*s*), 110.4 (*s*), 43.8 (*s*, CH_2_), 20.5 (*s*), 17.7 (*s*). Piperazine **2**: ^1^H NMR (CDCl_3_): δ 7.03 (*s*, 2H, aromatic CH), 7.01 (*s*, 4H, aromatic CH), 3.04 (*s*, 8H, CH_2_), 2.33 (*s*, 6H, CH_3_), 2.30 (*s*, 6H, CH_3_). ^13^C{^1^H} NMR (CDCl_3_): δ 149.5 (*s*), 132.9 (*s*), 132.8 (*s*), 132.0 (*s*), 127.2 (*s*), 119.3 (*s*), 52.8 (*s*), 20.9 (*s*), 18.0 (*s*). The procedure was based on that used for the fluoro analogue (Day *et al.*, 2011 ▸). Crystals of piperazine **2** were grown by slow evaporation of a toluene solution of the compound, at room temperature.

## Refinement details   

Crystal data, data collection and structure refinement details are summarized in Table 2 ▸. The N—H protons of compound **1** were located in the difference map and refined freely. The piperazine **2** crystallized in the non-centric group *P*2_1_; no heavy atoms are present in the structure, therefore the Flack parameter was not calculated. Carbon-bound hydrogen atoms were placed in calculated positions (C—H = 0.95–0.99 Å) and refined according to a riding model, with fixed *U*_iso_ values of 1.2 times (CH and CH_2_ groups) and 1.5 times (CH_3_ groups) the parent atom.

## Supplementary Material

Crystal structure: contains datablock(s) 1, 2. DOI: 10.1107/S2056989018011404/lh5879sup1.cif

Structure factors: contains datablock(s) 1. DOI: 10.1107/S2056989018011404/lh58791sup2.hkl

Structure factors: contains datablock(s) 2. DOI: 10.1107/S2056989018011404/lh58792sup3.hkl

CCDC references: 1861254, 1861253

Additional supporting information:  crystallographic information; 3D view; checkCIF report

## Figures and Tables

**Table 1:** Hydrogen-bond geometry (Å, °) for **1** [Chem scheme1]

*D*—H⋯*A*	*D*—H	H⋯*A*	*D*⋯*A*	*D*—H⋯*A*
N1—H1*A*⋯N2	0.80 (4)	2.44 (4)	3.172 (5)	154 (3)
N1—H1*B*⋯Br1	0.79 (4)	2.68 (4)	3.094 (4)	115 (4)
N2—H2*A*⋯N1^i^	0.81 (4)	2.43 (4)	3.155 (5)	149 (4)
N2—H2*B*⋯Br2	0.75 (4)	2.70 (4)	3.082 (4)	114 (3)

Symmetry code: (i) 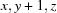.

**Table 2:** Experimental details

	C_8_H_10_BrN	C_20_H_26_N_2_
Crystal data		
*M*_r_	200.08	294.43
Crystal system, space group	Monoclinic, *P*2_1_/*c*	Monoclinic, *P*2_1_
Temperature (K)	150	150
*a*, *b*, *c* (Å)	16.4359 (10), 5.1917 (3), 20.5792 (11)	7.6563 (2), 13.2685 (4), 8.3688 (2)
β (°)	110.748 (4)	96.968 (2)
*V* (Å^3^)	1642.15 (17)	843.89 (4)
*Z*	8	2
Radiation type	Mo *K*α	Mo *K*α
μ (mm^−1^)	4.93	0.07
Crystal size (mm)	0.28 × 0.15 × 0.08	0.28 × 0.15 × 0.08

Data collection		
Diffractometer	Bruker APEXII CCD	Bruker APEXII CCD
Absorption correction	Numerical (*SADABS*; Bruker, 2015 ▸)	Numerical (*SADABS*; Bruker, 2015 ▸)
*T*_min_, *T*_max_	0.542, 0.746	0.894, 0.954
No. of measured, independent and observed [*I* > 2σ(*I*)] reflections	17969, 2903, 2170	13424, 3289, 2588
*R*_int_	0.052	0.074
(sin θ/λ)_max_ (Å^−1^)	0.595	0.625

Refinement		
*R*[*F* ^2^ > 2σ(*F* ^2^)], *wR*(*F* ^2^), *S*	0.030, 0.069, 1.00	0.040, 0.095, 1.05
No. of reflections	2903	3289
No. of parameters	201	203
No. of restraints	0	1
H-atom treatment	H atoms treated by a mixture of independent and constrained refinement	H-atom parameters constrained
Δρ_max_, Δρ_min_ (e Å^−3^)	0.45, −0.38	0.20, −0.19

Computer programs: *APEX2* and *SAINT* (Bruker, 2015 ▸), *SHELXT* (Sheldrick, 2015*a* ▸), *SHELXL* (Sheldrick, 2015*b* ▸) and *OLEX2* (Dolomanov *et al.*, 2009 ▸).

## supplementary crystallographic information

### 2-Bromo-4,6-dimethylaniline (1) Crystal data

**Table:**

C_8_H_10_BrN	*F*(000) = 800
*M**_r_* = 200.08	*D*_x_ = 1.619 Mg m^−^^3^
Monoclinic, *P*2_1_/*c*	Mo *K*α radiation, λ = 0.71073 Å
*a* = 16.4359 (10) Å	Cell parameters from 6401 reflections
*b* = 5.1917 (3) Å	θ = 2.7–27.3°
*c* = 20.5792 (11) Å	µ = 4.93 mm^−^^1^
β = 110.748 (4)°	*T* = 150 K
*V* = 1642.15 (17) Å^3^	Prism, colourless
*Z* = 8	0.28 × 0.15 × 0.08 mm

### 2-Bromo-4,6-dimethylaniline (1) Data collection

**Table:**

Bruker APEXII CCD diffractometer	2170 reflections with *I* > 2σ(*I*)
φ and ω scans	*R*_int_ = 0.052
Absorption correction: numerical (SADABS; Bruker, 2015)	θ_max_ = 25.0°, θ_min_ = 2.7°
*T*_min_ = 0.542, *T*_max_ = 0.746	*h* = −19→19
17969 measured reflections	*k* = −6→6
2903 independent reflections	*l* = −24→24

### 2-Bromo-4,6-dimethylaniline (1) Refinement

**Table:**

Refinement on *F*^2^	Primary atom site location: dual
Least-squares matrix: full	Hydrogen site location: mixed
*R*[*F*^2^ > 2σ(*F*^2^)] = 0.030	H atoms treated by a mixture of independent and constrained refinement
*wR*(*F*^2^) = 0.069	*w* = 1/[σ^2^(*F*_o_^2^) + (0.0241*P*)^2^ + 3.2572*P*] where *P* = (*F*_o_^2^ + 2*F*_c_^2^)/3
*S* = 1.00	(Δ/σ)_max_ = 0.001
2903 reflections	Δρ_max_ = 0.45 e Å^−^^3^
201 parameters	Δρ_min_ = −0.38 e Å^−^^3^
0 restraints	

### 2-Bromo-4,6-dimethylaniline (1) Special details

**Table:**

Geometry. All esds (except the esd in the dihedral angle between two l.s. planes) are estimated using the full covariance matrix. The cell esds are taken into account individually in the estimation of esds in distances, angles and torsion angles; correlations between esds in cell parameters are only used when they are defined by crystal symmetry. An approximate (isotropic) treatment of cell esds is used for estimating esds involving l.s. planes.

### 2-Bromo-4,6-dimethylaniline (1) Fractional atomic coordinates and isotropic or equivalent isotropic displacement parameters (Å^2^)

**Table:**

	*x*	*y*	*z*	*U*_iso_*/*U*_eq_	
Br1	0.97945 (2)	0.20330 (8)	0.56141 (2)	0.03211 (12)	
N1	0.8334 (2)	0.6135 (7)	0.52086 (17)	0.0268 (7)	
H1A	0.815 (2)	0.756 (8)	0.5140 (19)	0.020 (11)*	
H1B	0.871 (3)	0.593 (8)	0.505 (2)	0.035 (13)*	
C1	0.8515 (2)	0.5157 (6)	0.58759 (17)	0.0194 (7)	
C2	0.9120 (2)	0.3209 (7)	0.61459 (17)	0.0214 (8)	
C3	0.9268 (2)	0.2078 (7)	0.67863 (17)	0.0228 (8)	
H3	0.968810	0.074670	0.694815	0.027*	
C4	0.8800 (2)	0.2901 (7)	0.71895 (17)	0.0222 (7)	
C5	0.8196 (2)	0.4857 (7)	0.69261 (17)	0.0216 (8)	
H5	0.787150	0.543673	0.719926	0.026*	
C6	0.8041 (2)	0.6005 (7)	0.62857 (18)	0.0208 (8)	
C7	0.8930 (2)	0.1658 (8)	0.78885 (19)	0.0315 (9)	
H7A	0.881827	−0.019629	0.782470	0.047*	
H7B	0.852611	0.242654	0.808743	0.047*	
H7C	0.952953	0.194196	0.820356	0.047*	
C8	0.7372 (2)	0.8172 (7)	0.60306 (18)	0.0232 (8)	
H8A	0.766500	0.975665	0.597582	0.035*	
H8B	0.708709	0.845803	0.637008	0.035*	
H8C	0.693420	0.768935	0.558245	0.035*	
Br2	0.73280 (2)	0.70006 (8)	0.33028 (2)	0.03345 (12)	
N2	0.7421 (2)	1.1155 (7)	0.43964 (19)	0.0262 (7)	
H2A	0.745 (3)	1.262 (8)	0.454 (2)	0.026 (12)*	
H2B	0.771 (3)	1.104 (8)	0.419 (2)	0.024 (12)*	
C9	0.6585 (2)	1.0176 (7)	0.40744 (17)	0.0217 (8)	
C10	0.6405 (2)	0.8241 (7)	0.35792 (17)	0.0220 (8)	
C11	0.5595 (2)	0.7119 (7)	0.32828 (17)	0.0254 (8)	
H11	0.550493	0.580042	0.294449	0.030*	
C12	0.4915 (2)	0.7929 (7)	0.34820 (18)	0.0264 (8)	
C13	0.5085 (2)	0.9868 (7)	0.39782 (17)	0.0237 (8)	
H13	0.462454	1.043560	0.412055	0.028*	
C14	0.5893 (2)	1.1007 (7)	0.42741 (17)	0.0218 (8)	
C15	0.4027 (2)	0.6683 (8)	0.3184 (2)	0.0364 (10)	
H15B	0.371137	0.740538	0.272303	0.055*	
H15C	0.370039	0.701668	0.349132	0.055*	
H15A	0.409549	0.482129	0.314386	0.055*	
C16	0.6042 (2)	1.3165 (6)	0.48222 (16)	0.0191 (7)	
H16C	0.652570	1.268841	0.524484	0.029*	
H16A	0.551292	1.339629	0.493238	0.029*	
H16B	0.618051	1.477773	0.463684	0.029*	

### 2-Bromo-4,6-dimethylaniline (1) Atomic displacement parameters (Å^2^)

**Table:**

	*U*^11^	*U*^22^	*U*^33^	*U*^12^	*U*^13^	*U*^23^
Br1	0.0301 (2)	0.0367 (2)	0.0358 (2)	0.00185 (18)	0.01951 (18)	−0.00371 (18)
N1	0.0294 (19)	0.0256 (19)	0.0247 (17)	−0.0004 (16)	0.0088 (16)	0.0017 (15)
C1	0.0179 (18)	0.0172 (18)	0.0196 (18)	−0.0040 (14)	0.0024 (15)	0.0002 (14)
C2	0.0168 (17)	0.0228 (19)	0.0256 (19)	−0.0034 (16)	0.0089 (15)	−0.0073 (16)
C3	0.0155 (16)	0.0221 (18)	0.0278 (19)	0.0014 (15)	0.0041 (15)	0.0003 (16)
C4	0.0169 (17)	0.0249 (18)	0.0232 (18)	−0.0065 (16)	0.0053 (14)	−0.0025 (16)
C5	0.0162 (17)	0.0264 (19)	0.0228 (18)	−0.0023 (15)	0.0078 (15)	−0.0085 (16)
C6	0.0161 (17)	0.0178 (17)	0.0258 (19)	−0.0015 (15)	0.0042 (15)	−0.0035 (16)
C7	0.029 (2)	0.037 (2)	0.030 (2)	−0.0012 (18)	0.0115 (17)	0.0041 (18)
C8	0.0238 (18)	0.0161 (17)	0.0259 (18)	−0.0007 (16)	0.0040 (15)	−0.0055 (16)
Br2	0.0361 (2)	0.0404 (2)	0.0305 (2)	0.00730 (19)	0.02002 (18)	0.00411 (18)
N2	0.0225 (18)	0.0237 (19)	0.0312 (19)	−0.0051 (15)	0.0081 (16)	−0.0007 (16)
C9	0.0233 (19)	0.0205 (19)	0.0193 (18)	0.0011 (15)	0.0050 (15)	0.0071 (15)
C10	0.027 (2)	0.0234 (19)	0.0193 (17)	0.0047 (16)	0.0126 (15)	0.0056 (16)
C11	0.031 (2)	0.0235 (19)	0.0196 (17)	−0.0008 (17)	0.0069 (16)	−0.0017 (16)
C12	0.0242 (19)	0.0282 (19)	0.0230 (18)	0.0005 (17)	0.0037 (15)	0.0060 (17)
C13	0.0226 (19)	0.027 (2)	0.0226 (19)	0.0063 (16)	0.0097 (15)	0.0062 (16)
C14	0.0257 (19)	0.0193 (17)	0.0185 (17)	0.0037 (15)	0.0052 (15)	0.0030 (15)
C15	0.029 (2)	0.042 (3)	0.033 (2)	−0.0064 (19)	0.0049 (18)	0.000 (2)
C16	0.0192 (17)	0.0139 (17)	0.0210 (17)	0.0037 (15)	0.0031 (14)	0.0038 (15)

### 2-Bromo-4,6-dimethylaniline (1) Geometric parameters (Å, º)

**Table:**

Br1—C2	1.912 (3)	Br2—C10	1.910 (3)
N1—H1A	0.80 (4)	N2—H2A	0.81 (4)
N1—H1B	0.79 (4)	N2—H2B	0.75 (4)
N1—C1	1.394 (4)	N2—C9	1.394 (5)
C1—C2	1.390 (5)	C9—C10	1.386 (5)
C1—C6	1.406 (5)	C9—C14	1.407 (5)
C2—C3	1.383 (5)	C10—C11	1.382 (5)
C3—H3	0.9500	C11—H11	0.9500
C3—C4	1.384 (5)	C11—C12	1.385 (5)
C4—C5	1.389 (5)	C12—C13	1.390 (5)
C4—C7	1.521 (5)	C12—C15	1.513 (5)
C5—H5	0.9500	C13—H13	0.9500
C5—C6	1.386 (5)	C13—C14	1.382 (5)
C6—C8	1.531 (5)	C14—C16	1.546 (5)
C7—H7A	0.9800	C15—H15B	0.9800
C7—H7B	0.9800	C15—H15C	0.9800
C7—H7C	0.9800	C15—H15A	0.9800
C8—H8A	0.9800	C16—H16C	0.9800
C8—H8B	0.9800	C16—H16A	0.9800
C8—H8C	0.9800	C16—H16B	0.9800
			
H1A—N1—H1B	111 (4)	H2A—N2—H2B	108 (4)
C1—N1—H1A	117 (3)	C9—N2—H2A	116 (3)
C1—N1—H1B	115 (3)	C9—N2—H2B	115 (3)
N1—C1—C6	120.5 (3)	N2—C9—C14	120.8 (3)
C2—C1—N1	122.1 (3)	C10—C9—N2	122.1 (3)
C2—C1—C6	117.3 (3)	C10—C9—C14	117.0 (3)
C1—C2—Br1	118.8 (3)	C9—C10—Br2	118.6 (3)
C3—C2—Br1	117.9 (3)	C11—C10—Br2	118.0 (3)
C3—C2—C1	123.3 (3)	C11—C10—C9	123.4 (3)
C2—C3—H3	120.2	C10—C11—H11	120.2
C2—C3—C4	119.5 (3)	C10—C11—C12	119.6 (3)
C4—C3—H3	120.2	C12—C11—H11	120.2
C3—C4—C5	117.7 (3)	C11—C12—C13	117.7 (3)
C3—C4—C7	121.0 (3)	C11—C12—C15	120.9 (3)
C5—C4—C7	121.4 (3)	C13—C12—C15	121.4 (3)
C4—C5—H5	118.3	C12—C13—H13	118.5
C6—C5—C4	123.4 (3)	C14—C13—C12	123.0 (3)
C6—C5—H5	118.3	C14—C13—H13	118.5
C1—C6—C8	120.5 (3)	C9—C14—C16	120.0 (3)
C5—C6—C1	118.8 (3)	C13—C14—C9	119.3 (3)
C5—C6—C8	120.7 (3)	C13—C14—C16	120.7 (3)
C4—C7—H7A	109.5	C12—C15—H15B	109.5
C4—C7—H7B	109.5	C12—C15—H15C	109.5
C4—C7—H7C	109.5	C12—C15—H15A	109.5
H7A—C7—H7B	109.5	H15B—C15—H15C	109.5
H7A—C7—H7C	109.5	H15B—C15—H15A	109.5
H7B—C7—H7C	109.5	H15C—C15—H15A	109.5
C6—C8—H8A	109.5	C14—C16—H16C	109.5
C6—C8—H8B	109.5	C14—C16—H16A	109.5
C6—C8—H8C	109.5	C14—C16—H16B	109.5
H8A—C8—H8B	109.5	H16C—C16—H16A	109.5
H8A—C8—H8C	109.5	H16C—C16—H16B	109.5
H8B—C8—H8C	109.5	H16A—C16—H16B	109.5

### 2-Bromo-4,6-dimethylaniline (1) Hydrogen-bond geometry (Å, º)

**Table:**

*D*—H···*A*	*D*—H	H···*A*	*D*···*A*	*D*—H···*A*
N1—H1*A*···N2	0.80 (4)	2.44 (4)	3.172 (5)	154 (3)
N1—H1*B*···Br1	0.79 (4)	2.68 (4)	3.094 (4)	115 (4)
N2—H2*A*···N1^i^	0.81 (4)	2.43 (4)	3.155 (5)	149 (4)
N2—H2*B*···Br2	0.75 (4)	2.70 (4)	3.082 (4)	114 (3)

Symmetry code: (i) *x*, *y*+1, *z*.

### 1,4-Bis(2,4-dimethylphenyl)piperazine (2) Crystal data

**Table:**

C_20_H_26_N_2_	*F*(000) = 320
*M**_r_* = 294.43	*D*_x_ = 1.159 Mg m^−^^3^
Monoclinic, *P*2_1_	Mo *K*α radiation, λ = 0.71073 Å
*a* = 7.6563 (2) Å	Cell parameters from 4924 reflections
*b* = 13.2685 (4) Å	θ = 2.5–31.6°
*c* = 8.3688 (2) Å	µ = 0.07 mm^−^^1^
β = 96.968 (2)°	*T* = 150 K
*V* = 843.89 (4) Å^3^	Block, clear colourless
*Z* = 2	0.28 × 0.15 × 0.08 mm

### 1,4-Bis(2,4-dimethylphenyl)piperazine (2) Data collection

**Table:**

Bruker APEXII CCD diffractometer	2588 reflections with *I* > 2σ(*I*)
φ and ω scans	*R*_int_ = 0.074
Absorption correction: numerical (SADABS; Bruker, 2015)	θ_max_ = 26.4°, θ_min_ = 2.9°
*T*_min_ = 0.894, *T*_max_ = 0.954	*h* = −9→9
13424 measured reflections	*k* = −16→16
3289 independent reflections	*l* = −10→10

### 1,4-Bis(2,4-dimethylphenyl)piperazine (2) Refinement

**Table:**

Refinement on *F*^2^	Primary atom site location: dual
Least-squares matrix: full	Hydrogen site location: inferred from neighbouring sites
*R*[*F*^2^ > 2σ(*F*^2^)] = 0.040	H-atom parameters constrained
*wR*(*F*^2^) = 0.095	*w* = 1/[σ^2^(*F*_o_^2^) + (0.0509*P*)^2^] where *P* = (*F*_o_^2^ + 2*F*_c_^2^)/3
*S* = 1.05	(Δ/σ)_max_ = 0.001
3289 reflections	Δρ_max_ = 0.20 e Å^−^^3^
203 parameters	Δρ_min_ = −0.19 e Å^−^^3^
1 restraint	

### 1,4-Bis(2,4-dimethylphenyl)piperazine (2) Special details

**Table:**

Geometry. All esds (except the esd in the dihedral angle between two l.s. planes) are estimated using the full covariance matrix. The cell esds are taken into account individually in the estimation of esds in distances, angles and torsion angles; correlations between esds in cell parameters are only used when they are defined by crystal symmetry. An approximate (isotropic) treatment of cell esds is used for estimating esds involving l.s. planes.

### 1,4-Bis(2,4-dimethylphenyl)piperazine (2) Fractional atomic coordinates and isotropic or equivalent isotropic displacement parameters (Å^2^)

**Table:**

	*x*	*y*	*z*	*U*_iso_*/*U*_eq_	
N1	0.8569 (2)	0.56722 (15)	0.4969 (3)	0.0224 (5)	
N2	0.6207 (2)	0.45272 (16)	0.6642 (3)	0.0220 (5)	
C1	0.9201 (3)	0.4876 (2)	0.6112 (3)	0.0246 (6)	
H1A	1.040068	0.504463	0.662098	0.029*	
H1B	0.926270	0.423048	0.552878	0.029*	
C2	0.7987 (3)	0.4762 (2)	0.7399 (3)	0.0259 (6)	
H2A	0.841706	0.421380	0.814845	0.031*	
H2B	0.797117	0.539467	0.802411	0.031*	
C3	0.6774 (3)	0.5442 (2)	0.4243 (3)	0.0258 (6)	
H3A	0.676846	0.480219	0.363429	0.031*	
H3B	0.634424	0.598445	0.348234	0.031*	
C4	0.5577 (3)	0.5352 (2)	0.5544 (4)	0.0268 (6)	
H4A	0.557386	0.599282	0.614919	0.032*	
H4B	0.435857	0.521120	0.505348	0.032*	
C5	0.4986 (3)	0.41979 (19)	0.7690 (3)	0.0222 (6)	
C6	0.3523 (3)	0.3616 (2)	0.7052 (3)	0.0209 (6)	
C7	0.2394 (3)	0.3254 (2)	0.8102 (3)	0.0234 (6)	
H7	0.140547	0.286476	0.767276	0.028*	
C8	0.2643 (3)	0.3435 (2)	0.9749 (3)	0.0252 (6)	
C9	0.4095 (3)	0.4009 (2)	1.0347 (4)	0.0302 (7)	
H9	0.430569	0.414541	1.146814	0.036*	
C10	0.5236 (3)	0.4385 (2)	0.9336 (3)	0.0275 (6)	
H10	0.621291	0.478073	0.977545	0.033*	
C11	0.3192 (3)	0.3355 (2)	0.5289 (3)	0.0264 (6)	
H11A	0.254747	0.271661	0.515405	0.040*	
H11B	0.431892	0.328848	0.485378	0.040*	
H11C	0.249530	0.389040	0.471108	0.040*	
C12	0.1416 (4)	0.3004 (2)	1.0852 (4)	0.0350 (7)	
H12A	0.024784	0.290661	1.025118	0.052*	
H12B	0.132978	0.346997	1.174735	0.052*	
H12C	0.187303	0.235416	1.127501	0.052*	
C13	0.9794 (3)	0.59108 (19)	0.3864 (3)	0.0212 (6)	
C14	1.1194 (3)	0.65758 (19)	0.4351 (3)	0.0216 (6)	
C15	1.2329 (3)	0.6837 (2)	0.3236 (3)	0.0231 (6)	
H15	1.325513	0.729848	0.355421	0.028*	
C16	1.2165 (3)	0.64531 (19)	0.1680 (4)	0.0251 (6)	
C17	1.0792 (3)	0.5787 (2)	0.1236 (3)	0.0275 (6)	
H17	1.065047	0.550702	0.018299	0.033*	
C18	0.9624 (3)	0.5526 (2)	0.2311 (3)	0.0274 (6)	
H18	0.868867	0.507338	0.197703	0.033*	
C19	1.1465 (3)	0.7012 (2)	0.6018 (4)	0.0296 (7)	
H19A	1.210157	0.652630	0.675487	0.044*	
H19B	1.032038	0.715946	0.637638	0.044*	
H19C	1.215095	0.763545	0.601050	0.044*	
C20	1.3401 (3)	0.6774 (2)	0.0498 (4)	0.0307 (7)	
H20A	1.290904	0.736048	−0.011167	0.046*	
H20B	1.354809	0.621855	−0.024576	0.046*	
H20C	1.454590	0.695166	0.108346	0.046*	

### 1,4-Bis(2,4-dimethylphenyl)piperazine (2) Atomic displacement parameters (Å^2^)

**Table:**

	*U*^11^	*U*^22^	*U*^33^	*U*^12^	*U*^13^	*U*^23^
N1	0.0194 (10)	0.0218 (12)	0.0258 (12)	0.0019 (8)	0.0017 (9)	0.0032 (10)
N2	0.0184 (10)	0.0228 (12)	0.0238 (12)	0.0004 (8)	−0.0005 (9)	0.0036 (10)
C1	0.0180 (11)	0.0238 (13)	0.0311 (17)	0.0023 (10)	−0.0007 (10)	0.0038 (12)
C2	0.0213 (11)	0.0271 (15)	0.0284 (16)	−0.0012 (10)	−0.0010 (10)	0.0042 (13)
C3	0.0204 (12)	0.0288 (14)	0.0276 (15)	0.0024 (10)	0.0003 (10)	0.0061 (12)
C4	0.0188 (12)	0.0265 (14)	0.0344 (17)	0.0025 (10)	0.0011 (11)	0.0062 (13)
C5	0.0200 (12)	0.0220 (14)	0.0244 (16)	0.0035 (9)	0.0015 (10)	0.0031 (11)
C6	0.0198 (12)	0.0211 (13)	0.0213 (14)	0.0044 (9)	0.0004 (10)	0.0006 (11)
C7	0.0195 (12)	0.0223 (14)	0.0276 (16)	−0.0006 (10)	0.0000 (10)	−0.0009 (12)
C8	0.0258 (12)	0.0217 (14)	0.0287 (16)	0.0004 (10)	0.0060 (11)	−0.0009 (12)
C9	0.0348 (14)	0.0343 (16)	0.0214 (15)	−0.0017 (12)	0.0031 (12)	−0.0067 (13)
C10	0.0266 (13)	0.0244 (14)	0.0306 (16)	−0.0053 (11)	0.0003 (11)	−0.0030 (12)
C11	0.0222 (12)	0.0295 (15)	0.0269 (16)	−0.0016 (11)	0.0004 (11)	−0.0008 (13)
C12	0.0395 (15)	0.0368 (18)	0.0297 (18)	−0.0049 (12)	0.0085 (13)	−0.0013 (14)
C13	0.0171 (11)	0.0190 (13)	0.0271 (16)	0.0027 (9)	0.0012 (10)	0.0026 (11)
C14	0.0192 (11)	0.0221 (14)	0.0227 (15)	0.0038 (10)	−0.0010 (10)	0.0013 (11)
C15	0.0208 (12)	0.0207 (13)	0.0264 (16)	−0.0017 (10)	−0.0025 (11)	0.0002 (11)
C16	0.0241 (13)	0.0235 (14)	0.0270 (16)	0.0029 (10)	−0.0003 (11)	0.0027 (12)
C17	0.0301 (14)	0.0291 (15)	0.0227 (15)	−0.0014 (11)	0.0006 (11)	−0.0064 (12)
C18	0.0228 (12)	0.0275 (15)	0.0308 (17)	−0.0056 (11)	−0.0012 (11)	−0.0030 (12)
C19	0.0266 (12)	0.0358 (17)	0.0261 (16)	−0.0042 (11)	0.0026 (11)	−0.0036 (13)
C20	0.0318 (14)	0.0351 (17)	0.0259 (17)	−0.0037 (12)	0.0065 (12)	−0.0024 (13)

### 1,4-Bis(2,4-dimethylphenyl)piperazine (2) Geometric parameters (Å, º)

**Table:**

N1—C1	1.466 (3)	C9—C10	1.381 (4)
N1—C3	1.466 (3)	C10—H10	0.9500
N1—C13	1.431 (3)	C11—H11A	0.9800
N2—C2	1.465 (3)	C11—H11B	0.9800
N2—C4	1.472 (3)	C11—H11C	0.9800
N2—C5	1.426 (3)	C12—H12A	0.9800
C1—H1A	0.9900	C12—H12B	0.9800
C1—H1B	0.9900	C12—H12C	0.9800
C1—C2	1.514 (4)	C13—C14	1.410 (3)
C2—H2A	0.9900	C13—C18	1.388 (4)
C2—H2B	0.9900	C14—C15	1.393 (4)
C3—H3A	0.9900	C14—C19	1.502 (4)
C3—H3B	0.9900	C15—H15	0.9500
C3—C4	1.511 (4)	C15—C16	1.390 (4)
C4—H4A	0.9900	C16—C17	1.390 (4)
C4—H4B	0.9900	C16—C20	1.511 (4)
C5—C6	1.411 (3)	C17—H17	0.9500
C5—C10	1.390 (4)	C17—C18	1.387 (4)
C6—C7	1.391 (4)	C18—H18	0.9500
C6—C11	1.507 (4)	C19—H19A	0.9800
C7—H7	0.9500	C19—H19B	0.9800
C7—C8	1.389 (4)	C19—H19C	0.9800
C8—C9	1.390 (4)	C20—H20A	0.9800
C8—C12	1.508 (4)	C20—H20B	0.9800
C9—H9	0.9500	C20—H20C	0.9800
			
C3—N1—C1	109.8 (2)	C5—C10—H10	119.3
C13—N1—C1	112.98 (18)	C9—C10—C5	121.4 (2)
C13—N1—C3	115.7 (2)	C9—C10—H10	119.3
C2—N2—C4	109.2 (2)	C6—C11—H11A	109.5
C5—N2—C2	116.4 (2)	C6—C11—H11B	109.5
C5—N2—C4	114.96 (19)	C6—C11—H11C	109.5
N1—C1—H1A	109.5	H11A—C11—H11B	109.5
N1—C1—H1B	109.5	H11A—C11—H11C	109.5
N1—C1—C2	110.71 (19)	H11B—C11—H11C	109.5
H1A—C1—H1B	108.1	C8—C12—H12A	109.5
C2—C1—H1A	109.5	C8—C12—H12B	109.5
C2—C1—H1B	109.5	C8—C12—H12C	109.5
N2—C2—C1	109.4 (2)	H12A—C12—H12B	109.5
N2—C2—H2A	109.8	H12A—C12—H12C	109.5
N2—C2—H2B	109.8	H12B—C12—H12C	109.5
C1—C2—H2A	109.8	C14—C13—N1	119.1 (2)
C1—C2—H2B	109.8	C18—C13—N1	122.0 (2)
H2A—C2—H2B	108.2	C18—C13—C14	118.9 (2)
N1—C3—H3A	109.7	C13—C14—C19	121.6 (2)
N1—C3—H3B	109.7	C15—C14—C13	118.4 (2)
N1—C3—C4	109.7 (2)	C15—C14—C19	119.9 (2)
H3A—C3—H3B	108.2	C14—C15—H15	118.6
C4—C3—H3A	109.7	C16—C15—C14	122.9 (2)
C4—C3—H3B	109.7	C16—C15—H15	118.6
N2—C4—C3	109.1 (2)	C15—C16—C20	121.2 (2)
N2—C4—H4A	109.9	C17—C16—C15	117.6 (3)
N2—C4—H4B	109.9	C17—C16—C20	121.2 (3)
C3—C4—H4A	109.9	C16—C17—H17	119.6
C3—C4—H4B	109.9	C18—C17—C16	120.8 (3)
H4A—C4—H4B	108.3	C18—C17—H17	119.6
C6—C5—N2	119.0 (2)	C13—C18—H18	119.3
C10—C5—N2	122.2 (2)	C17—C18—C13	121.4 (2)
C10—C5—C6	118.7 (2)	C17—C18—H18	119.3
C5—C6—C11	121.8 (2)	C14—C19—H19A	109.5
C7—C6—C5	118.4 (2)	C14—C19—H19B	109.5
C7—C6—C11	119.7 (2)	C14—C19—H19C	109.5
C6—C7—H7	118.5	H19A—C19—H19B	109.5
C8—C7—C6	123.1 (2)	H19A—C19—H19C	109.5
C8—C7—H7	118.5	H19B—C19—H19C	109.5
C7—C8—C9	117.4 (2)	C16—C20—H20A	109.5
C7—C8—C12	121.4 (2)	C16—C20—H20B	109.5
C9—C8—C12	121.2 (3)	C16—C20—H20C	109.5
C8—C9—H9	119.5	H20A—C20—H20B	109.5
C10—C9—C8	121.0 (3)	H20A—C20—H20C	109.5
C10—C9—H9	119.5	H20B—C20—H20C	109.5
